# Cardiomyopathy as the first manifestation of Friedreich's ataxia

**DOI:** 10.4322/acr.2020.204

**Published:** 2020-09-10

**Authors:** Rafael Tuzino Leite Neves Maffei, Giulio de los Santos Fortuna, Luca Campolino Rosso, Pedro Dragone Pires, Ivan Rondelli

**Affiliations:** 1 Irmandade da Santa Casa de Misericórdia de São Paulo, Faculdade de Ciências Médicas da Santa Casa de São Paulo, Departamento de Anatomia Patológica, São Paulo, SP, Brasil

**Keywords:** Friedreich Ataxia, Cerebellar Ataxia, Cardiomyopathies, Autopsy

## Abstract

We present the case of a female patient diagnosed in childhood with Friedreich Ataxia (FA). At the age of 6, she developed left congestive heart failure with cardiomyopathy, as evident on echocardiogram. Neurologic signs only appeared at age 7, including marked loss of muscle mass, gait instability, muscle clonus, and Babinski's signal. At age 27, she had a stroke and was hospitalized; a few days later, she had a cardiorespiratory arrest with asystole, leading to death. The autopsy disclosed severe cardiomyopathy and significant myocardial replacement with fibrosis; therefore, the cause of death was assumed to be heart failure. Compared to the literature, our case has some unique features, such as cardiac disease as the presenting manifestation instead of gait instability, which is the major initial sign in most FA cases. Since our patient was submitted to an autopsy, it was an opportunity to retrieve important data to confirm the diagnosis and to evaluate the pathophysiology of this entity, such as myocardium fibrosis and cerebellar degeneration. In summary, our case demonstrates that cardiac disease can be the first manifestation of FA, with eventual diagnostic and prognostic implications. In addition, the autopsy provided findings of severe cardiomyopathy associated with FA.

## INTRODUCTION

In 1863, Nikolaus Friedreich (1825-1882) was the first to describe early-onset hereditary ataxia associated with kyphoscoliosis and degeneration of the heart.^1^ The disease is due to an autosomal recessive mutation of the *FRDA* gene on chromosome 9q13, which is responsible for frataxin expression. This protein’s insufficiency is suggested to be associated with a variety of molecular disruptions and cell death, with mitochondrial iron accumulation and elevated levels of reactive oxygen species. These events result in gradual cell damage and, eventually, cell death. This cell injury occurs mainly in the neurons, resulting in neuronal depletion, and heart cells, leading to fibrosis.^2^ ^-^ ^6^

Friedreich ataxia (FA) is the most common inherited ataxia, with an estimated incidence of 1:29,000 in Caucasians. The clinical features are progressive and multisystemic, with the first signs and symptoms appearing in adolescence. Gait instability is usually the first manifestation, followed by peripheral sensory neuropathy, vestibular changes, hyporeflexia, myoclonus, and dysarthria. Systemic presentations are scoliosis, cardiomyopathy, diabetes mellitus, and foot deformities such as cavus foot.^7^ ^,^ ^8^ When affected by the disease, the myocardium is hypertrophic with thick ventricular walls; it generally maintains adequate systolic functions, but may progress to heart failure and death.^9^

## CASE REPORT

Our patient is a female who was diagnosed with FA in childhood using the diagnostic criteria from the Quebec Cooperative Study on Friedreich’s Ataxia: onset at ≤ 20 years, progressive ataxia, lower limb areflexia, decreased vibration sense, weakness, and dysarthria. These diagnostic criteria, when present, provide 63% sensitivity and 98% positive predictive value.^10^ ^,^ ^11^ Genetic testing was not available at the time. At age 3, she was diagnosed with a heart murmur, and exertional dyspnea ensued. Three years later, she developed the clinical features of congestive heart failure, and cardiomyopathy was diagnosed on echocardiogram. The exam showed hypertrophy and dilation of the left ventricle with diffuse hypokinesia. Neurologic signs started to show at age 7, including marked loss of muscle mass, gait instability, muscle clonus, and Babinski's signal. She also rapidly developed progressive scoliosis (above 60 degrees), which was surgically treated at the age of 13. Her neurologic disease deteriorated rapidly, and, at age 10, she was already experiencing frequent falls and dysarthria, and lost the lower limb deep reflexes.

There was no family history of neurological or heart diseases. At age 27, she had a stroke and was hospitalized. At this time, her left ventricle had severe diffuse hypokinesia and an ejection fraction of 32% on echocardiogram. Mild eccentric biventricular dilation was found, but there were no signs of pulmonary hypertension. The exam identified laminar pericardial effusion but did not detect significant valve changes. Five days later, she had a cardiorespiratory arrest with asystole, which lead to death.

## AUTOPSY FINDINGS

**Figure 1:**
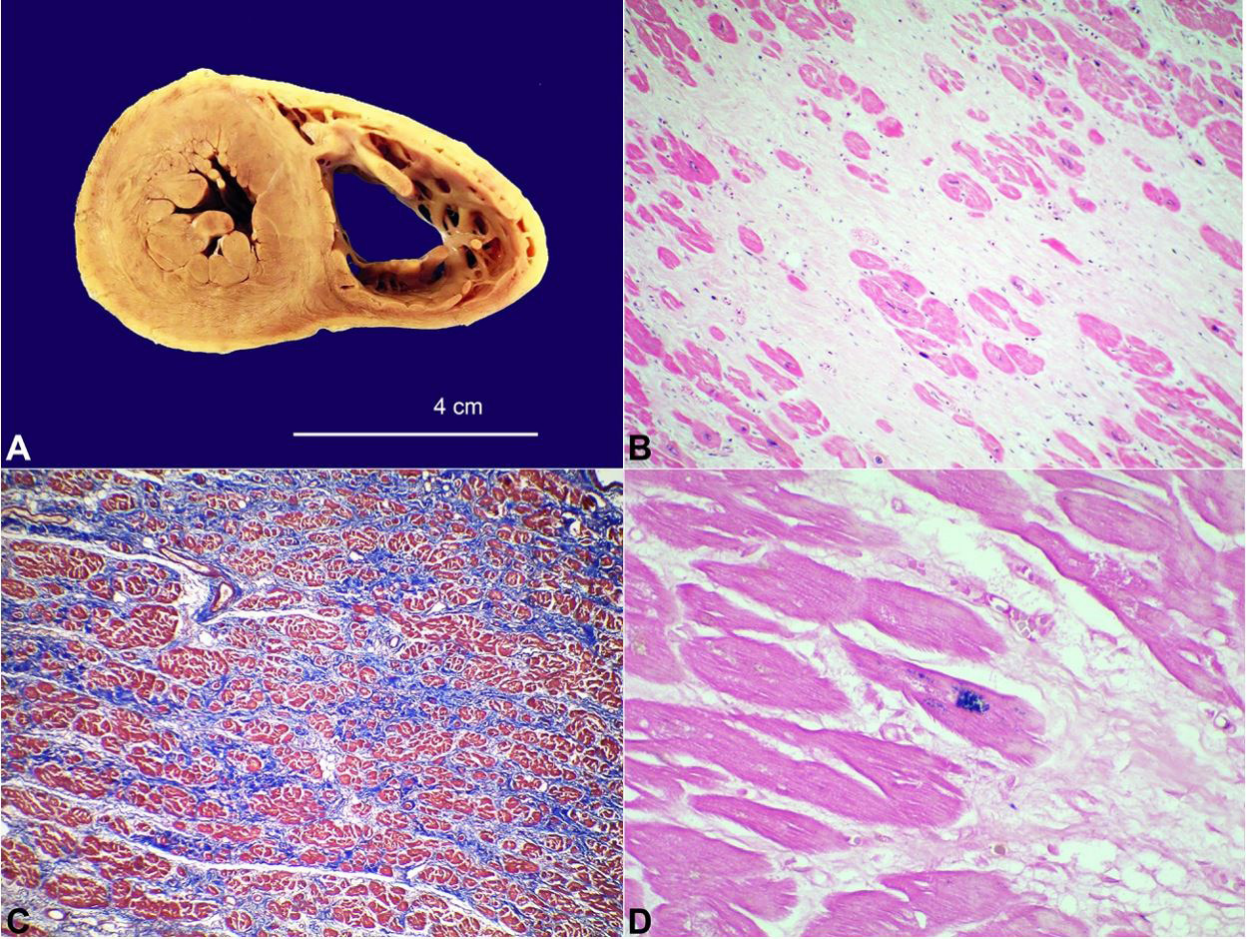
**A –** Gross view of the heart, showing the pale appearance of the left heart and dilation of the right ventricle. (B, C, and D, photomicrographs of the heart.). **B –** Myocardial replacement by fibrosis (H&E, 100X optical magnification OM). **C –** Remaining myocardiocytes showed in red and fibrosis replacing myocardium in blue (Masson's Trichrome Stain 40X OM). **D –** The blue spots indicate iron storage in the cardiac fibers (Perls Prussian Blue Stain, 400X OM).

An autopsy was performed less than 24 hours post-mortem and disclosed heart failure as the cause of death. The heart weighed 350 grams (reference range [RR] in women; 312 ± 78 g.),^12^ with a smooth and translucent pericardium. Once the cavities were open, the most relevant finding was dilation of the right ventricle, with thin walls, of 0.2 cm (RR: 0.4–0.6 cm). The wall thickness of the left ventricle and the septum was 1.2 cm (RR: 1.1–1.5 cm).^13^ The myocardium was firm, with small yellowish areas in the region of the right ventricular outflow tract (Figure 1A). Examination of the valves and coronaries showed no changes. Microscopic examination showed significant myocardial replacement with fibrosis (Figures 11C) and a mural thrombus at the tip of the left ventricle. In addition, multiple foci of iron deposition were detected in cardiomyocytes (Figure 1D). The iron deposits were not found in other organs, such as the central nervous system (CNS), spleen, and liver. Examination of the lungs showed edema and hemorrhagic areas, which were compatible with acute heart failure, and a focal area of pneumonia. No signs of chronic pulmonary hypertension were found.

**Figure 2:**
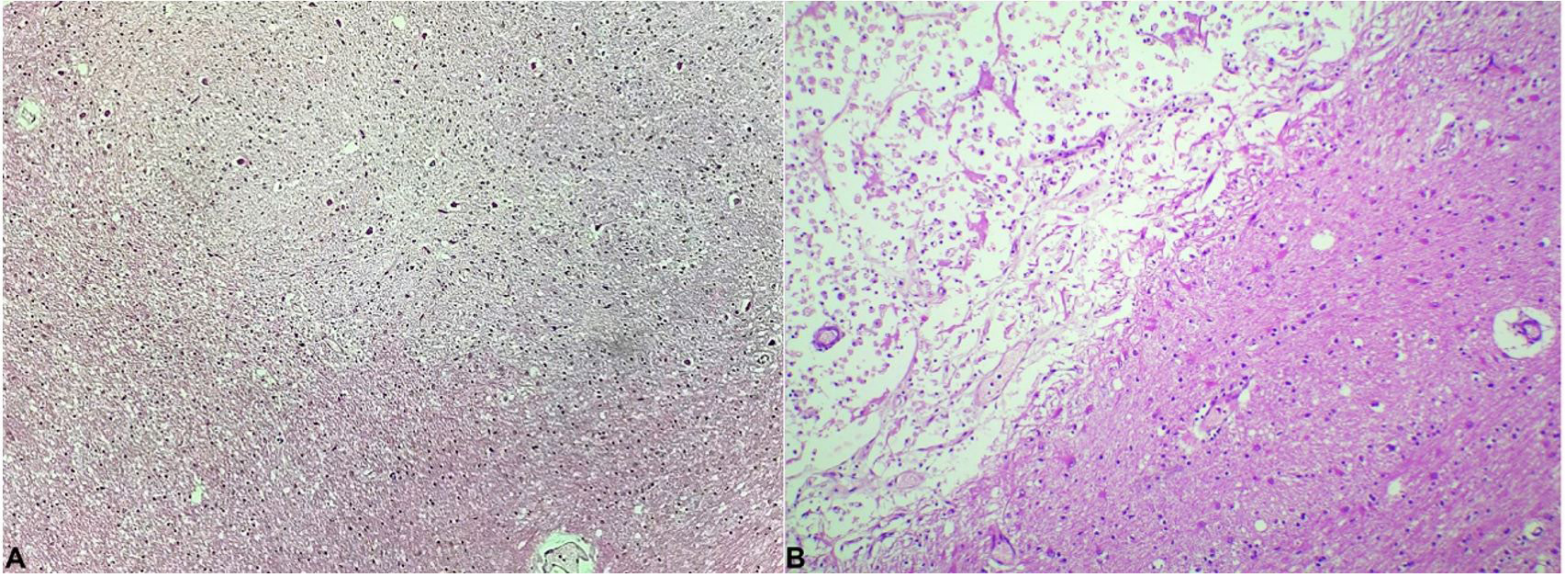
Photomicrographs of the central nervous system. **A –** Cerebellum with neuronal depletion and discrete gliosis of the dentate nucleus (H&E, 100X OM). **B –** Medial temporal lobe showing infarction with xanthomized macrophages (upper left) (H&E, 100X OM).

CNS examination demonstrated marked spinal cord atrophy—mainly in the dorsal spinal column-and cerebellar degeneration, with scant cellularity in the deep cerebellar nuclei and reactive gliosis (Figure 2A). A focal area of ischemia was also detected in the temporal lobe represented by a softened area on the medial temporal lobe projecting to the lentiform nucleus on gross pathology. Recent infarction was confirmed on histopathology (Figure 2B). This finding was compatible with the clinical stroke and may be related to the left ventricle thrombus.

No changes were found in the anatomopathological examination of the pancreas, liver, spleen, and gastrointestinal tract. Histological examination of the kidneys showed mild acute tubular necrosis and anemic infarction, attributed to the patient’s critical hemodynamic state during hospitalization.

## DISCUSSION

Our case has some unique features compared to the literature. First, cardiac disease was the presenting manifestation, as opposed to gait instability, which was the most common initial sign in 78% of patients in the cohort of the European FA Consortium for Translational Studies. In this group, cardiac manifestations were detected in only 5% of patients when first evaluated.

Second, our patient had progressive and severe cardiac disease compared with most patients with FA, who usually develop neurologic symptoms only at age 9–19 years and develop cardiac manifestations later in life.^14^ ^-^ ^19^ Early cardiac manifestations may be related to a worse outcome of the disease and are usually the leading cause of death.^20^ ^,^ ^21^

Finally, the autopsy performed in our case provided important data to confirm the diagnosis and to establish anatomoclinical correlation, as demonstrated by myocardium fibrosis and cerebellar degeneration. The FA-mutated *FRDA* gene is involved in the transcription of frataxin, a mitochondrial protein with homeostasis function in iron metabolism. Although the exact role of frataxin in the development of FA is not known, it is hypothesized that deficiency of this enzyme causes cellular oxidative stress and iron storage.^22^ ^-^ ^25^

The iron accumulation in tissues of FA patients is described. It is more associated with the heart, which is commonly reported as multifocal (as in our case) and, in some cases, the liver and spleen. Although frataxin deficiency is reported in several different tissues, its surplus in the heart is not yet well understood, but it may be related to greater mitochondrial function and generation of oxidative stress in cardiomyocytes.^26^ ^-^ ^29^ Although genetic testing may demonstrate the mutation in the frataxin gene, this was not done, in our case, because we were unable to extract adequate material from the paraffin block. Nonetheless, this was not deemed necessary for the diagnosis of FA, due to the typical clinical and anatomopathological findings.^30^ ^,^ ^31^

## CONCLUSION

Our case demonstrates that cardiac disease may be the first manifestation of FA, perhaps with diagnostic and prognostic implications. It also provides autopsy findings of severe cardiomyopathy in this disease.
